# Physician assistant education program director attrition and consideration to leave

**DOI:** 10.1186/s12909-023-04640-3

**Published:** 2023-09-03

**Authors:** Cody A. Sasek, Jonathan L. Kilstrom, Sebastian Opar, Zachary Simons

**Affiliations:** 1 Butler University Doctor of Medical Science Bridge Program, Indianapolis, IN USA; 2 Yale University PA Online Program, New Haven, CT USA; 3 Carbon Health Urgent Care, Green Brook, NJ USA; 4 Schweiger Dermatology Group, Hamburg, NY USA

**Keywords:** Physician assistant education, Burnout, Leadership, Attrition, Faculty development

## Abstract

**Background:**

This study explores factors related to physician assistant (PA) education program directors’ (PD) consideration to leave their leadership role. This is important to better understand, with the need for additional PA education PDs as the number of PA programs grows in addition to current PA program leaders considering leaving their PD role.

**Methods:**

Data from the 2019 Physician Assistant Education Association (PAEA) Faculty and Directors Survey were used to analyze factors related to consideration for leaving the PD position. Multiple logistic regression analyses were utilized to identify predictors of PD consideration for leaving their position. Multiple regression analyses were also used to explore factors related to burnout.

**Results:**

The study found burnout was a modest predictor for consideration of leaving the PD position, while underrepresented minority status was not. Additional job stress, job satisfaction, and job experience variables were found to have a modest relationship with consideration of leaving, with odds ratios between 0.28 (lack of faculty respecting each other) and 5.29 (stress from lack of personal time) for those with statistically significant relationships.

**Conclusions:**

PD consideration of leaving is a complex phenomenon with many variables and confounding factors likely at play, including, as demonstrated by this study, level of burnout. Study implications include a further understanding of how effective strategies might be designed and implemented to address the drivers of PA PD attrition. Further exploration of burnout as a possible mediating variable as well as more specific data collection directed at better understanding predictors of PD attrition would be valuable future research directions.

## Background

A physician assistant/associate (PA) is a licensed healthcare clinician. PAs practice medicine across specialties and settings, taking medical histories, conducting physical exams, diagnosing and treating illness, ordering and interpreting diagnostic tests, developing treatment plans, and prescribing medications. Admission to PA training programs requires an earned bachelor’s degree. Accepted students are conferred a master’s degree after completion of approximately 28 months of classroom instruction and clinical rotations [1]. The number of physician assistant (PA) education programs has grown considerably in the last decade, with 326 programs projected by 2024 [2]. These trends have led to a resultant need for additional PA education program directors (PDs). At the same time, there is concerning data regarding current PA PDs’ consideration for leaving the PD role.

PA PDs provide PA educational programs with operational, curricular, and personnel leadership. They must be a current or emeritus certified PA and have at least three years of full-time higher education experience. PDs are expected to prepare and submit accreditation reports, provide leadership to faculty, staff, and students, provide ongoing administrative leadership of daily operations, oversee the program’s finances and resources, and conduct continuous program review, analysis, planning, and development. Many institutions also require PDs to contribute scholarly activity and have clinical appointments [3]. PA PDs are often provided a stipend for their administrative duties in addition to a base salary. Median total compensation for PA PDs of $131,000 was 1.7% lower in 2019 than the $133,000 earned by their clinical practice counterparts with similar levels of experience (15–19 years) [4, 5].

The Accreditation Review Commission on Education for the Physician Assistant (ARC-PA) indicated that from 2018 to 2022, the biannual rate of PD changes ranged between 4.94 and 10.10% [6]. The total number of PD changes, not including those with appointments to newly developed programs during that time was 174 [6]. This was also a time in which the number of PA programs grew from 239 to 300 [2]. The reported level of attrition, along with increased need for PDs, has led to concern about the ability to maintain an adequate supply of PA PDs.

In the most recent Physician Assistant Education Association (PAEA) survey data from 2019, 43.1% of PA faculty and 47.3% of PDs reported they were considering leaving academia for another job, with the majority of respondents in both categories reporting feelings of burnout a few times a month or more [4]. PDs report self-imposed high expectations, increased work responsibilities, and institutional procedures and “red tape” as the top three most stressful aspects of the position [4].

Research specific to PA education has demonstrated a relationship between job satisfaction, burnout, and turnover [7–10]. Burnout has been identified as a contributor to faculty consideration to leave their position in PA faculty generally (AOR 1.97, p < .001) as well as more specifically in clinical directors (AOR 2.70, p < .001), who had nearly twice the odds of considering leaving their program (95% CI, 1.34–2.71) for each 1-point increase in burnout on a 7-point scale [11].

The aim of this study is to explore the relationship of factors related to consideration of leaving the PD position for PA education program PDs. Factors explored in the current study included those related to job experiences, job satisfaction, job stress, burnout, and underrepresented minority status.

The literature has demonstrated success in several PA PD leadership development efforts [12–15]. More must be known about drivers of consideration to leave PA education leadership roles, so that strategies can be effectively designed and implemented in those areas most impactful to the development and retention of PA PDs.

## Methods

This study was a secondary analysis of data from the PAEA-developed and administered 2019 PAEA Faculty and Directors Survey. The survey dataset was obtained after a request to PAEA. In addition to demographic information, the PAEA-developed survey included study outcome measures focused on consideration of leaving the PD position and survey items exploring perceptions of fairness in the PA program, job satisfaction, stressors, and burnout. Within these broad categories, all individual survey items were included in the data analysis to allow for the most specific exploration of factors possible within the bounds of available survey data. The data was requested as germane to the research question and also best able to provide a variety of dimensions from which to explore PA PD’s consideration for leaving the role.

A total of 211 PD responses were obtained from the 243 accredited programs at that time which were contacted to participate. From the 211 PD responses, 10 were removed due to incomplete responses for the majority of survey items. Deletion of incomplete responses ultimately led to a PD response rate of 82.7%.

As noted, total responses from those identifying as PDs (n = 211) included several with missing item responses. For respondents with more than three non-responses to items (n = 10, 4.7%), listwise deletion was applied to those participants’ data. For respondents with less than three non-responses in the 61-item survey, imputation of those missing variables was accomplished with the use of series mean values. Following data imputation, paired t-tests identified no absolute differences in mean or standard deviation values of all items.

To conduct a secondary analysis of survey data, multiple logistic regressions were utilized to identify the presence of predictors for PD consideration of leaving their position. Respondent consideration of leaving their position was based on three survey items asking participants to identify whether, in the last two years, they had considered: Leaving academia, leaving their PD position for another position at the same institution, or leaving the institution.

Further analysis of the strength of association of burnout with possible predictors of leaving the PD position was carried out with multiple regression analyses. Burnout was classified by using a survey item asking respondents to rate the frequency of burnout on a seven-point scale from Never to Every day.

Finally, analyses of the relationship of underrepresented minority status (those who identified as Hispanic, a single non-White race, or a non-White race in combination with White race) to consideration for leaving variables and burnout was conducted through logistic regression and student’s t-test, respectively.

The institutional review boards of Yale University and Butler University confirmed the project as not in need of IRB review. All analyses were performed with SPSS Statistics for Windows, Version 26.0 (IBM Corp.).

## Results

### Participant characteristics

Table 1 shows the demographic characteristics of survey respondents. Response totals vary between categories as some responses were omitted or multiple options were selected, where applicable. For statistical analyses, underrepresented minority classification included any respondent who identified as Hispanic, a single non-White race, or a non-White race in combination with White race.

**Table 1:** Demographics of 2019 PAEA program director survey respondents

*Age mean = 51.3; n = 201*			
		n	%
Gender Identity			
	Male	83	39.3
	Female	123	58.3
	Prefer not to answer	5	2.4
	Other*	0	0.0
	Total	211	100.0
Race			
	American Indian or Alaskan Native	0	0 0.0
	Asian	3	1 0.4
	Black or African American	11	5 0.3
	Multiracial	6	2 0.9
	Native Hawaiian or other Pacific Islander	0	0 0.0
	White or European American	177	84 0.7
	Other	4	1 0.9
	Prefer not to answer	8	3 0.8
	Total	209	100.0
Ethnicity			
	Hispanic, Latino, Latina, or Spanish in origin	9	4 0.3
	Not Hispanic, Latino, Latina, or Spanish in origin	193	92 0.3
	Prefer not to answer	7	3 0.3
	Total	209	100.0
Underrepresented Minority (URM)			
	URM	28	14.1
	Non-URM	170	85.9
	Total	198	100.0
Time in PA Education			
	Less than 1 year	0	0 0.0
	1 year	4	1 0.9
	2–4 years	28	13 0.6
	5–9 years	56	27 0.2
	10–14 years	41	19 0.9
	15 or more years	77	37 0.4
	Total	206	100.0
Highest Degree Currently Held			
	Bachelor’s Degree	0	0 0.0
	Master’s Degree	114	54 0.5
	Doctoral Degree	95	45 0.5
	Total	209	100.0

*Other includes: Indigenous or other cultural gender minority (e.g., two-spirit) and Something else (e.g., gender fluid, non-binary)

### Impact of job stress, satisfaction, and experiences on consideration for leaving

During the past two years, 43.8% (n = 88) of PDs reported receiving at least one firm job offer elsewhere, with 43.8% (88) having considered leaving the institution for another institution, 47.3% (95) having considered leaving academia for another job, and 24.4% (49) having considered leaving their current position for another role in the same PA program. Analysis of job stress, job satisfaction, and job experiences survey items as predictors of consideration for leaving variables identified several variables with statistically significant relationships, with stress from lack of personal time being greatest (OR 5.29, *P* < .001). These variables are summarized in Table 2.

Of note, data analysis exploring PD consideration for leaving did not identify the presence of statistically significant relationships between study outcome consideration of leaving variables and work environment, faculty development both internally and externally, fairness of salary, salary amount, perception of teaching workload, value of teaching, value of research, value of service, or stress of expectations.

**Table 2:** Statistically significant predictors of PD consideration of leaving

	Independent Variable	Sig	Exp(B)	95% C.I. for Exp(B)	
				Lower	Upper
Considered leaving to another institution	Lack of faculty respecting each other	0.01	0.28	0.12	0.68
	Lack of satisfaction with curriculum	0.05	2.08	1.00	4.32
	Lack of satisfaction with institutional leadership	0.01	2.15	1.26	3.66
	Colleagues as a source of stress	0.05	1.83	1.01	3.31
	Review/promotion process as source of stress	0.01	2.13	1.17	3.88
	Stress from workplace discrimination	0.05	2.56	1.00	6.55
Considered leaving role, staying with same institution	Lack of satisfaction with job duties	< 0.001	4.06	1.86	8.85
	Colleagues as a source of stress	0.03	2.15	1.09	4.27
	Increased work responsibilities	0.02	2.96	1.23	7.14
	Institutional procedures and “red tape”	0.05	0.46	0.22	0.99
Considered leaving academia	Lack of preparation for conflict over diversity issues in the classroom	0.03	1.66	1.05	2.62
	Lack of satisfaction with clinical work arrangement	0.04	0.65	0.43	0.98
	Lack of satisfaction with job duties	0.03	2.30	1.07	4.93
	Lack of satisfaction with quality of students	0.02	0.41	0.20	0.89
	Stress from lack of job security	0.02	2.43	1.19	4.99
	Stress from lack of personal time	< 0.001	5.29	2.42	11.57
	Stress from managing household responsibilities	0.04	0.52	0.27	0.98

### Burnout and consideration for leaving

Burnout was found to have a statistically significant relationship with consideration for leaving variables (leaving academia, leaving the PD position for another at the same institution, and leaving the institution: all *P* < .001), with modest associated odds ratios (ORs 1.48–1.65). Table 3 contains results from logistic regression of the impact of self-reported burnout frequency on consideration for leaving respondents’ current PD position. Table 4 includes the statistically significant results from multiple regression analyses of burnout and consideration of leaving.

**Table 3:** Relationship of burnout to PD consideration of leaving

	B	Sig	Exp(B)	95% C.I. for Exp(B)	
				Lower	Upper
Leave Institution	0.39	< 0.001	1.48	1.23	1.79
Leave Role, Same Institution	0.44	< 0.001	1.56	1.25	1.94
Leave Academia	0.50	< 0.001	1.65	1.36	2.01

**Table 4:** Statistically significant relationships of job experience, stress, and satisfaction variables to burnout

	B	S.E.	t-stat	Sig	Tolerance	VIF
Satisfaction with institutional leadership	0.29	0.15	2.00	0.048	0.34	2.91
Increased work responsibilities	0.80	0.19	4.23	< 0.001	0.40	2.48
Stress from lack of personal time	0.56	0.21	2.73	0.007	0.39	2.59
Stress from self-imposed high expectations	0.41	0.17	2.39	0.018	0.57	1.77
Lack of administrator consideration for faculty concerns	0.39	0.15	2.57	0.011	0.39	2.60

### URM Status, consideration for leaving, and burnout

Analysis of the impact of underrepresented minority status on consideration for leaving outcomes identified no statistically significant (*P* < .05) relationships between the variables: Considering to leave the institution for another institution (*P* = .49; 95% CI 0.59-3.01); leaving the role but staying in the institution (*P* = .27; 95% CI 0.17-1.64); or leaving academia (*P* = .30; CI 0.68 − 3.50). Additional analysis of the relationship of URM status to burnout identified equality of group variances with Levene’s Test significance value of 0.187. A lower mean burnout score was found for URM respondents (3.41) than non-URM respondents (3.92), although there was no statistically significant relationship between degree of burnout and URM status (t= -1.507, *P* = .134).

## Discussion

PDs play a crucial role in ensuring future PAs receive requisite training and education. However, evidence suggests rates of turnover for this position are high [6]. To address the issue, the current study identified several factors related to PD consideration for leaving. This appears to be the first such study to focus specifically on this topic. Recent investigations of this study’s question have been related to other populations of PA faculty, including those in a clinical education director role [16] and Graham’s [17] qualitative exploration of PA faculty in general.

The current rate of attrition [6], coupled with a rising demand for PDs [2] signals a concern related to the sufficiency of the supply of qualified PA PDs. Consistent with the related literature, the current study found a significant relationship of burnout, job factors, and elements of job satisfaction to PA PD consideration for leaving.

The findings of the current study align with research exploring other populations of PA faculty. For clinical education directors, Klein et al [16]. highlighted similar findings to the current study, with connections of burnout (*P* < .001) and dissatisfaction with professional development (*P* < .001) to intention to leave. Clinical directors with high burnout scores had dramatically increased odds of intent to leave academia (AOR = 2.7, 95% CI: 1.80–4.05; *P* = < 0.001).^16^ For PA faculty in a variety of roles who had left a faculty position, inadequate faculty development was found to be a dominant theme [17].

To address the issue of maintaining an adequate supply of PA PDs, a comprehensive and proactive approach should be considered, in line with recommendations from the work of Beltyukova and Graham [18], which included recognition by administration, support for scholarly work, support of the PA program by administration, a fair promotion process, and a sense of institutional community.

First, efforts targeted at reducing and preventing burnout can be made to enhance the job satisfaction of current PDs by providing opportunities for professional development, mentorship, and leadership training. The perceived value and retention benefits of professional development for PA faculty have been described in the literature [19, 20], including those specific to PA PDs [21].

Second, strategies can be employed to attract and retain new talent to PA education. This can include offering competitive compensation packages, promoting a positive work-life balance, and providing a supportive work environment. Although compensation was not shown to impact consideration for leaving in the current study, competitive salaries may help recruit PAs to education leadership roles.

As previously noted, burnout has been identified as a potential mediating variable for attrition. To gain further insight, it would be worthwhile to conduct more specific research that delves into the impact of burnout on PDs’ perception of job-related factors, such as their experiences, stressors, satisfaction, and the likelihood of considering leaving their role. By analyzing the relationship between burnout and these variables using a validated instrument, such as the Maslach Burnout Inventory, further study could offer insight into the complex interplay of factors that influence PD attrition and mitigate a limitation of the current study. A clearer understanding of these dynamics would further support administrators’ investment in strategies to prevent burnout and improve PD well-being, thereby likely reducing turnover rates and improving organizational performance.

An additional limitation of the current study is its use of secondary data, particularly with the framing of the outcome as subjective consideration of leaving variables, rather than a more objective outcome, such as having left the PD position. A specifically designed data collection instrument and/or qualitative approach could help more precisely understand the experience of PDs. The current study utilized the most recent available data, although the data was collected prior to the COVID-19 pandemic. Similar analysis of more recent PD-specific data would help clarify the impact of the pandemic on PA PD consideration for leaving the PD position and related attrition. This is important as Neary and colleagues [22] found PDs had the greatest increase in stress among PA faculty as a result of the pandemic.

## Conclusion

Current study findings are valuable to inform strategies aimed at mitigating the negative effects of PD attrition and related implications for the quality of education and training of future PAs. An exploration of current PA education data raises concerns about the adequacy of the supply of qualified PA PDs. This study has demonstrated a relationship between burnout, job experience factors, job satisfaction factors, and consideration for leaving the PD position. To address this issue, effective strategies must be designed and implemented to address the drivers of PA PD attrition.

## Data Availability

The data that support the findings of this study are available from the Physician Assistant Education Association, but restrictions apply to the availability of these data, which were used under license for the current study, and so are not publicly available. Data are however available from the authors upon reasonable request and with permission of Physician Assistant Education Association.

## Abbreviations

PA: Physician Assistant
PD: Program director
